# A Review of THz Technologies for Rapid Sensing and Detection of Viruses including SARS-CoV-2

**DOI:** 10.3390/bios11100349

**Published:** 2021-09-22

**Authors:** Naznin Akter, Muhammad Mahmudul Hasan, Nezih Pala

**Affiliations:** Department of Electrical and Computer Engineering, Florida International University, Miami, FL 33174, USA; nakte001@fiu.edu (N.A.); mhasa043@fiu.edu (M.M.H.)

**Keywords:** SARS-CoV-2, terahertz, plasmonics, biosensing, metasenors, immunobiosensing

## Abstract

Virus epidemics such as Ebola virus, Zika virus, MERS-coronavirus, and others have wreaked havoc on humanity in the last decade. In addition, a coronavirus (SARS-CoV-2) pandemic and its continuously evolving mutants have become so deadly that they have forced the entire technical advancement of healthcare into peril. Traditional ways of detecting these viruses have been successful to some extent, but they are costly, time-consuming, and require specialized human resources. Terahertz-based biosensors have the potential to lead the way for low-cost, non-invasive, and rapid virus detection. This review explores the latest progresses in terahertz technology-based biosensors for the virus, viral particle, and antigen detection, as well as upcoming research directions in the field.

## 1. Introduction

A rare incidence of pneumonia was identified in China in late 2019 [1], which was later determined to be caused by a novel severe acute respiratory syndrome β-coronavirus (SARS-CoV-2) or novel coronavirus [2]. The World Health Organization (WHO) classified this unique coronavirus disease (COVID-19) as a pandemic after it spread rapidly around the world [3]. There have been 220,563,227 confirmed cases of COVID-19 recorded to WHO as of 6 September 2021, with 4,565,483 deaths and a total of 5,352,927,296 vaccine doses delivered [4]. After severe acute respiratory syndrome (SARS) in 2003 [5] and Middle East Respiratory Syndrome (MERS) in 2012 [6,7,8], COVID-19 is the third large-scale pandemic produced by a coronavirus in the previous two decades. In the case of SARS-CoV-2, the number of laboratory-confirmed COVID-19 infections has now surpassed the total number of SARS and MERS cases by more than 90 times [9]. Despite the new Coronavirus, there are a number of other virus-related diseases that represent a considerable threat to the global population. Therefore, reliable, fast, easy-to-use, and inexpensive virus detection methods are needed to restrain the spread of diseases and preclude future pandemics like COVID-19.

The primary established viral diagnosis methods include CRISPR–Cas12-based detection [10], quantitative real-time polymerase chain reaction (RT-PCR) [11,12], enzyme-linked immunosorbent assay (ELISA) [13], and point-of-care (POC) lateral flow immunoassay test [14,15]. Although molecular techniques have been the standard method to detect the presence of viral genetic material in infected patients, it may produce false-negative results if viral RNA is insufficient at the time of detection [16]. Immunoassays [16,17], which use the spike (S), receptor-binding domain (RBD), and nucleocapsid (N) antigens and IgM, IgG, and IgA antibodies, can provide information on both ongoing viral infections and earlier exposures. The prime drawback of immunoassays is the failure to identify infection during the early stages of the disease, since antibodies take several days to form after contact with foreign material. In [18], a methodological notion for a cell-based biosensor technique has been proposed using membrane-engineered mammalian cells carrying the human chimeric spike S1 antibody. It can produce results within three minutes, with a detection limit of 1 fg/mL and a semi-linear response range of 10 fg to 1μg/mL, albeit clinical validation of the assay using patient samples has yet to be established. Most of these diagnosis processes suffer from some common limitations [16,19], which include: (i) long turnaround time; (ii) slow detection process; (iii) poor sensitivity; (iv) requirement of certified facilities; (v) need for sophisticated equipment; (vi) well-trained personal to handle the testing; (vii) invasive protocols; and (viii) expense. A detailed summary showing several advantages and disadvantages of these detection methods has been reported in [20].

Several works have recently been reported on plasmonic- and metamaterial-based plasmonic biosensors for virus or viral particle detection, as well as various methods of Coronavirus detection [16,21,22,23,24], and recent progress in nanophotonic biosensors to combat the COVID-19 pandemic [25]. Although they allow rapid detection with high sensitivity, plasmonic- and metamaterial-based biosensors have not yet become available for clinical use. Considering these available detection methods, Terahertz technology is ideal for addressing these existing detection problems and meeting the constant need for selective, repeatable, label-free, cost-effective, on-chip, ultrasensitive, and easy-to-use biosensors. Terahertz (THz) waves have low energy (~few meV) that is below the ionization energies of atoms and molecules, making it possible to analyze materials without disrupting the system under investigation. The hydrogen bond, the most prominent bond in biological molecules, has characteristic energy within the THz frequency range. Therefore, THz spectroscopy techniques may directly identify spectral properties such as resonances and rotational and vibrational motion of molecules and can be used to detect viruses.

Despite its immense potential, THz-based virus detection is still in its infancy. This paper reviews the existing THz-based virus sensing methods along with COVID-19 sensing and its promising outlook for future pathogenic virus detection to prevent any potential pandemic. The following is the outline of this paper: Section 2 briefly reviews SARS-CoV-2, its existing variants, and available detection methods. In Section 3, recent advances in virus sensing using terahertz spectroscopy and metamaterials are reviewed. Finally, SARS-CoV-2 detection using terahertz techniques in comparison with conventional methods is presented. The goal of this comprehensive review of terahertz technology for virus detection, including SARS-CoV-2, is to highlight the recent achievements and point out future research opportunities in the field.

## 2. SARS-CoV-2, Its Variants and Existing Diagnosis Methods

SARS-CoV-2 (severe acute respiratory syndrome coronavirus 2) is a virus that belongs to the coronavirus family that provokes respiratory illness in humans. It was transmitted from animals to humans in a mutated form and was first identified in December 2019 in Wuhan, China. The SARS-CoV-2 virus causes COVID-19, a disease named by the World Health Organization on 11 February 2020 [26]. Coronaviruses (CoVs) are enclosed positive-stranded RNA viruses and possess the largest RNA genome among all known viruses. They belong to the Coronaviridae family which has four primary genera (alpha, beta, gamma, and delta-CoVs) [27,28]. 

**Figure 1:**
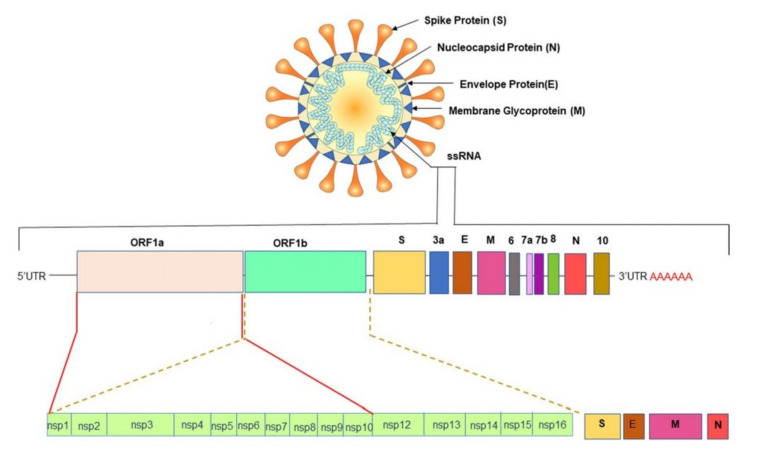
Schematic presentation of spherical genome structure of the SARS-CoV-2. Reprinted with permission from [29]. Copyright 2020 Springer.

All CoVs exhibit a similar structure with an identical genome order as shown in Figure 1 [29]. Inside the hemagglutinin esterase viral membrane, the viral genome and nucleocapsid protein (N) bind together to create a helical case. A nucleocapsid (N), a membrane protein (M), a spike protein (S), a small membrane envelope protein (E), and an internal protein (I) are all encoded by the viral gene [28]. One of the initial steps in coronavirus infection is the precise attachment of the coronavirus spike (S) protein to the cellular entry receptors. Several coronavirus receptors have been found [29], including human aminopeptidase N (APN; HCoV-229E), angiotensin-converting enzyme 2 (ACE2; HCoV-NL63, SARS-CoV and SARS-CoV-2), and dipeptidyl peptidase 4 (DPP4; HCoV-NL63, SARS-CoV and SARS-CoV-2) (DPP4; MERS-CoV). As a result, the appearance and the tissue orientation of entrance receptors affect the viral pathogenicity and tropism [30]. It is evident from scanning electron micrographs (SEM) that the coronavirus appears oval or spherical, similar to other coronaviridae family, with stalk-like projections ending in a round structure (spike). The viral particle size ranges from 60 to 140 nm [31,32] and its infectivity and host specificity are both dependent on spikes.

The untranslated regions (5′ and 3′) of large RNA genomes of Coronaviruses contain cis-acting secondary RNA structures which are required for RNA synthesis. Due to the nature of mRNA viruses to make complete duplicates or mutants that are merged into newly made viral particles (Figure 1), Coronaviruses reproduce and express their genomic RNA during their intracellular life cycle. Geographical separation also plays an essential role in producing genetically different variations. The importance of early detection of SARS-CoV-2 has been highlighted by its fast evolution and mutation. New information concerning these variations’ virologic, epidemiologic, and clinical properties is rapidly becoming available. SARS-CoV-2 variants have been identified as 20I/501Y.V1, VOC 202012/01, or B.1.1.7 in the United Kingdom (UK), 20H/501Y.V2 or B.1.351 in South Africa, P.1 in Brazil [33], and E484Q, L245R are two different mutations of B.1.617, which is a double mutant variant. There is also a triple mutant variant B1.618 in India [34,35,36]. Both double and triple mutant variants are now classified as variants of interest (VOIs). There are four distinct mutations in the spike protein of B.1.618 that are linked to enhanced infectivity and immunological escape. New genetic sets have been discovered in the new triple mutant corona virus and parts of the E484K version are also present in it. The triple mutant corona virus B.1.618 can compromise anyone’s immune system. Antibodies present in the bodies of people who have already been infected with the coronavirus are known as triple mutant corona virus deficient [34]. These new variants are creating considerable concerns due to their capacity to evade natural- or vaccine-induced immunity as well as existing therapies [37,38]. The new variants are also highly transmissible (70% more transmissible than the old variant), resistant, and carry an elevated risk of death. Efficient testing and detection methods are crucial to suppress the growing infection rate.

**Figure 2:**
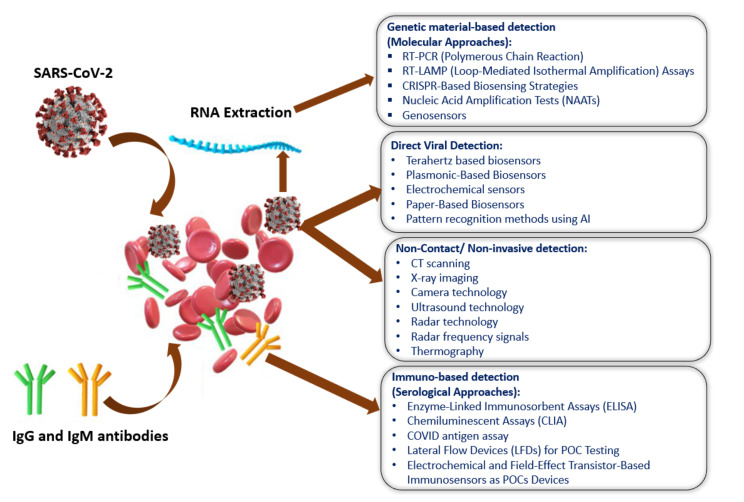
A visual summary of the currently available SARS-CoV-2 detection process. Reprinted with permission from [39]. Copyright 2020 American Chemical Society.

Figure 2 shows the current methods of SARS-CoV-2 detection, which have been categorized into four sections: (i) genetic material-based detection; (ii) direct viral detection; and (iii) non-invasive detection; and (iv) immuno-based detection [39]. The SARS-CoV-2 spike protein is the best option (among the four-protein spike, envelope, matrix, and nucleocapsid) to be used as a diagnostic antigen since it is highly immunogenic and is a key transmembrane protein of the virus. Moreover, the spike protein facilitates the SARS-CoV-2 detection process by revealing the sequence variation of amino acid among coronaviruses.

## 3. THz Techniques for Virus Sensing

Terahertz radiation is also referred to as submillimeter radiation. The term describes electromagnetic waves with frequencies ranging from 100 GHz in the millimeter-wave band to 10 THz in the far-infrared light spectrum. The frequency range of the THz wave correlates to the vibrational frequencies of numerous significant biomolecules (proteins, RNA, and DNA), allowing biomolecule vibration to be detected. THz technology is well known for its high penetration into most dielectrics, low photon energy (~4 meV @ 1 THz), unique molecular signatures, and non-ionizing nature which make it suitable for label-free and non-contact modalities for sensing and detection [40,41]. On the other hand, low spatial resolution is one of the main drawbacks of THz radiation, owing to its long wavelength. Lateral dimensions of a typical virus are less than 100 nanometers, making it difficult to spatially resolve using THz [42]. Furthermore, the high sensitivity of THz radiation to polar molecules, particularly water, restricts THz wave penetrability in hydrated materials to tens to hundreds of microns, posing a challenge for use in biological applications [40,43]. Such challenges led to the development of ingenious methods to respond and make THz available for biological sensing, detection, and imaging applications [44,45]. THz sensing methods are broadly based on two approaches: (1) measuring the shifts in the resonant frequencies of THz metamaterials due to the ambient refractive change caused by the virus loading. (2) Measuring the absorption spectrum to identify the peaks caused by the viruses. A combination of these could be used as well.

Metamaterials are artificial materials with electromagnetic properties do not present in nature [46], such as a negative index of refraction or electromagnetic cloaking. A metamaterial is made up of several unit cells or individual elements (sometimes called “meta-atoms”), each of which is much smaller than the wavelength with which it interacts. Traditional materials like metals and dielectrics are used to build these unit cells microscopically. However, their precise shape, geometry, size, orientation, and arrangement can have unusual macroscopical effects on light, such as generating resonances or exceptional macroscopic permittivity and permeability values. Metamaterials’ unique electromagnetic characteristics open the door to a wide range of THz applications. Metamaterials and metasurfaces are the most extensively employed method in terahertz technology to improve detection sensitivity while attaining sub-wavelength spatial resolution through high electromagnetic field confinement. Single- or multi-resonance frequencies of the THz metamaterials devices mostly depend on the unique geometry of unit cell and on the substrate refractive index and this resonance frequency may shift (red or blue shifted) based on the sample deposition. Substrate and metal properties influence the magnetic responses of metamaterials at terahertz regions. The sensitivity of metamaterials can be defined by the refractive index unit (RIU) and the virus surface number density unit, N_av_ [47].

(1) $$\mathrm{Sensitivity}\;\mathrm{in}\;\mathrm{refractive}\;\mathrm{index}\;\mathrm{unit}=\frac{\Delta f}{\Delta n}\left[\frac{\mathrm{GHz}}{\mathrm{RIU}}\right]$$

(2) $$\;\mathrm{Sensitivity}\;\mathrm{in}\;\mathrm{virus}\;\mathrm{surface}\;\mathrm{number}\;\mathrm{density}\;\mathrm{unit}=\frac{\Delta f}{N_{\mathrm{av}}}\left[\frac{\mathrm{GHz}.{µm}^{2}}{\mathrm{particle}}\right]$$

where,

(3) $$\mathrm{Virus}\;\mathrm{surface}\;\mathrm{number}\;\mathrm{density}\;\mathrm{unit},N_{\mathrm{av}}=\frac{N_{\mathrm{virus}}}{\mathrm{Area}}\;$$

The sensitivity and resonance response of metamaterials are highly dependent on the shapes and refractive index of the material. Sensitivity refers to the sensor’s level of reaction to external influences, as shown by Equations (1) and (2). When using a frequency shift sensing method, a high Q is needed for high sensitivity. Because, at the very least, a narrower peak can provide better resolution for shifting. The metamaterial unit cell area must be well designed to improve detection capabilities and the desired resonance peak. Biosensing with terahertz metamaterials does not involve sample preparation, does not damage the sample, gives accurate results, and has a short detection time.

**Figure 3:**
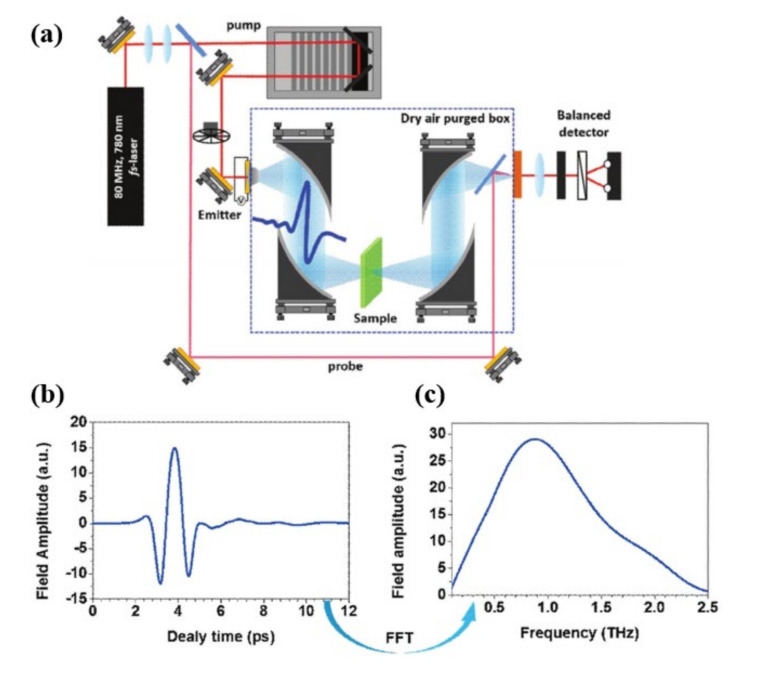
(**a**) Schematic of a standard THz-TDS setup, (**b**) time domain THz pulse, and (**c**) frequency domain THz pulse. Reprinted with permission from [48]. Copyright 2019 Wiley.

In THz biosensors, the analyte concentration is often measured using either transmission spectrum or absorption (intensity) spectrum approaches. A femtosecond pulsed laser is employed in a typical THz-TDS setup, as shown in Figure 3 [48]. Initially, a pulsed femtosecond laser is split into two beams: a pump beam for producing pulsed THz waves and probe beams for detecting THz waves that have been transmitted. A single cycle of a THz wave is produced by pump pulses impinging on an emission crystal (GaAs) based emitter with an antenna where a high voltage is provided. After passing via a guiding mirror, the generated THz waves collide with the separated probe beam in the detecting crystal (ZnTe). The time difference between the two pulse beams is used to acquire the time-based waveform trace the pulsed THz wave. The transmitted (reflected) THz beam to the sample is acquired once the target sample is positioned in the focused zone. The THz beam route and samples are tightly sealed and filled with dry air or nitrogen to avoid exposure to moisture in the air. The fast Fourier transform can convert time-based pulsed THz waveforms to frequency-based data (FFT), and the optical characteristics of sample materials can be determined using the following relationship. Figure 3b,c shows a typical time and frequency domain-based terahertz pulse spectrum. The transmission spectra, T, can be determined by measuring the transmitted electric field through the empty substrate and the electric field through the sample onto the substrate, as follows:

(4) $$T=\frac{I_{\mathrm{sample}}\;\left(\omega \right)}{I_{\mathrm{ref}}\left(\omega \right)}=\frac{E_{\mathrm{sample}}{\left(\omega \right)}^{2}}{E_{\mathrm{ref}}{\left(\omega \right)}^{2}}$$

where,

(5) $$E_{\mathrm{sample}}\left(\omega \right)={\;E}_{\mathrm{ref}}\left(\omega \right)\;\exp\left(-\frac{d\alpha \left(\omega \right)}{2}\right)\;\exp\left(i\frac{2\pi }{\lambda }\;n\left(\omega \right)d\right)$$

Equation (5) satisfies the absorption, α(ω), and the complex refractive index, n(ω), for the sample thickness, *d*. Terahertz wave absorption spectra in a material can also be represented by using the wavelength, λ and the imaginary component of the refractive index, κ.

(6) $$\alpha \left(\omega \right)=-\frac{1}{d}\ln\left(T\right)=-\frac{1}{d}\ln\left(\frac{E_{\mathrm{sample}}{\left(\omega \right)}^{2}}{E_{\mathrm{ref}}{\left(\omega \right)}^{2}}\right)=\frac{4\pi \kappa }{\lambda }$$

The absorption peak(s) appear(s) when robust vibration mode(s) is(are) at a specific frequency, and its(their) position and amplitude depend(s) on the sample’s molecular structure. Hence, the absorption spectrum is an extremely useful indicator for identifying and distinguishing the substance, allowing for THz spectroscopy-based sensing, and detecting applications due to its dependency on the unique molecular structure.

**Figure 4:**
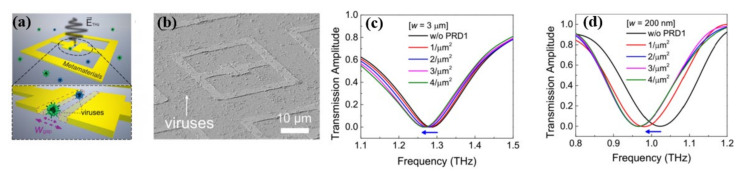
(**a**) Diagram of virus detection with THz nano-gap metamaterial. (**b**) Image (SEM) of viruses (width of gap 200 nm) and THz transmission amplitudes (normalized) of THz metamaterials with deposited PRD1 of different surface densities for gap width of (**c**) w = 3 µm (**d**) w = 200 nm. Reprinted with permission from [42]. Copyright 2019 The Optical Society.

One of the earliest reports on the use of THz for virus detection was by Park et al. [42] using THz-time domain spectroscopy (TDS) measurements and a nano-gap metamaterial (Figure 4a,b). Using a narrowly defined detection volume of metamaterials, the authors claimed to be able to sense the number of microorganisms in the narrow gap area as well as detect the target material with exceptional sensitivity. However, present THz metamaterial sensing research has been limited to micro-sized microorganisms (~λ/100) due to the similar dimensions of the common material gap and the targeted microorganisms. As a result, it is hard to manage the detection of tiny viruses which have a size of below 100 nm, and not much has been done in this area previously. Here, they have demonstrated the detection of PRD1 (60 nm) double-stranded DNA virus, bacteriophage viruses, and MS2 (30 nm) single-strand RNA virus. The shift in the resonant frequency of the metamaterials was observed following the deposition of viruses to analyze their potential utility in low-concentration viral detection. The surface density was regulated by the virus coating, and the major goal was to identify the smallest amount of single particle per μm^2^ in surface density. An increase in PRD1 sensitivity with reduced gap width was observed owing to the enhanced field localization. The THz transmission amplitudes of PRD1 and MS2 reveal that size matching can improve detection sensitivity for low surface densities, as illustrated in Figure 4c,d. The detection sensitivity for a gap width of w = 20 nm is 80 GHz·μm^2^/particle, but for w = 3 μm, it is 6 GHz·μm^2^/particle. The resonant frequency shifts when the effective dielectric constant changes in the gap area because of the deposited virus, which has a surface number density of 4/μm^2^ for PRD1 (Figure 4c). In the case of MS2 viruses, a similar but larger shift in frequency was observed.

**Figure 5:**
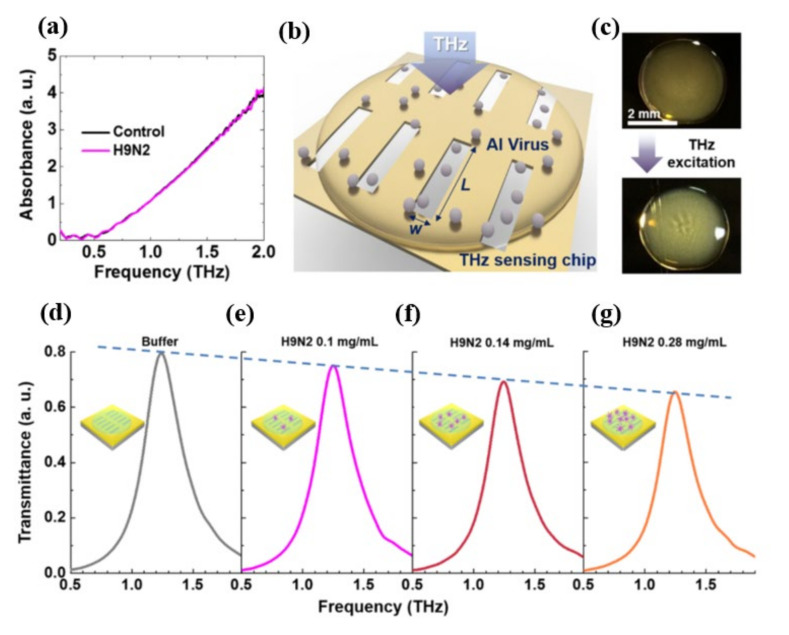
(**a**) Absorption spectra for pallet types of viruses included a protein sample (H9N2) and a control sample (without virus). (**b**) A conceptual schematic of THz detection of virus samples. (**c**) Optical images of dropped virus solutions onto the multi-resonance nano-antenna array before (top) and after (down) THz excitation. (**d**–**g**) Normalized THz spectra for various concentrations (0, 1, 0.14, 0.28 mg/mL) of H9N2 virus in buffer solution. Reprinted with permission from [49]. Copyright 2017 Springer.

Lee et al. [49] employed THz spectroscopy to investigate the THz optical characteristics of various types of Avian Influenza (AI) viruses inside the spectral range from 0.2 to 2 THz utilizing a nano-metamaterial sensing chip. The H9N2 virus and the control samples (without any virus inoculation) had no discernible absorption features in the THz spectrum when measured directly (Figure 5a). This was explained by inhomogeneous broadening of absorption and the superposition of multiple protein vibration mode characteristics [50], which implies that inadequate chemical specificity and low THz detection sensitivity are the key roadblocks to THz sensing applications in pathogenic monitoring. The nano-metamaterial sensing chip used in the study was based on the previous work on THz nano-antennas with a log-periodic alignment [51] and allowed for sensing of optically hidden target molecules in the ultra-broadband THz regime with very high sensitivity. THz transmittance measurement through the viral sample dropped sensing chip is shown in Figure 5b. 

As seen at the top of Figure 5c, the deposited sample of the virus forms a thin coating on the multi-resonance detecting chip. These nano-antennas were formed by gold with a thickness of 150 nm deposited on a 500-μm-thick double-side-polished silicon wafer. A whole area of 2 mm × 2 mm was designed with more than 1000 slots to reduce the probable errors which could occur during the liquid drop casting because of the random distribution of protein sample. Moreover, each viral sample (H1N1, H5N2) exhibits a distinct transmission spectrum shift as well as a distinct shift in resonance frequency as a result of their different THz absorption characteristics, which indicates the high selectivity potential. According to the authors, the proposed device may identify optically hidden bio specimens, such as viruses, without their distinctive fingerprinting within a dependable spectral span. The optical parameters of the obtained THz spectra for distinct viral samples, including absorption characteristics and complex refractive index, were investigated thoroughly and the tested viruses could be classified based on their subtypes. Furthermore, the viral quantification was successful in a concentration-dependent manner. The transmission spectra of the H9N2 virus were studied using a nano-antenna sensing chip to confirm that the concentration dependent mechanism might allow the THz sensing chip to identify the virus at different concentrations as shown in Figure 5d–g. Diluting viral samples with buffer liquid in a 1:1 and 2:1 volume ratio yielded four samples with different virus concentrations of 0, 1, 0.14, 0.28 mg/mL, respectively. The maximum transmittance value decreases as the virus concentration rises due to a change in absorption.

**Figure 6:**
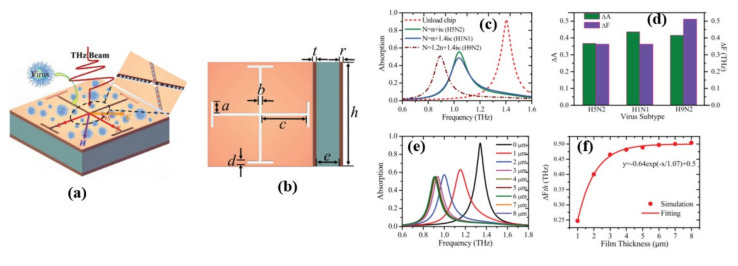
(**a**) 3D schematic diagram of the THz biosensing metamaterial absorber (**b**) geometrical parameters, (**c**) FDTD simulation results of absorption spectroscopy for three equivalent AI virus model samples: n + iκ (H5N2), n + 1.4iκ (H1N1), 1.2n + 1.4iκ (H9N2), (**d**) ΔA and ΔF of three different AI virus model samples: *n* + iκ (H5N2), *n* + 1.4iκ (H1N1), 1.2n + 1.4iκ (H9N2), (**e**) FDTD simulation results of absorption spectroscopy with analyte thickness for H9N2, (**f**) ΔFth versus different virus thickness of H9N2. Reprinted with permission from [52]. Copyright 2018 Wiley.

Metamaterial absorbers with Jerusalem cross apertures has also been used for detecting various subtypes and sensing the protein concentration of AI viruses based on the strongly confined spoof surface plasmon polaritons (SSPPs) resonance mode in the THz region [52]. The resolution of field–matter interaction features within a strongly confined resonance field and sharp spectral features can be improved by using a metamaterial biosensing absorber [53,54,55,56], which can improve biosensing sensitivity for biological materials and has a wide range of applications in medical imaging, early disease prediction, DNA/RNA analysis, and time-sensitive virus biosensing [42,49,57,58]. In the study, equivalent complex refractive index (N) of the selected protein concentrations and virus subtypes were used. The proposed THz biosensing metamaterial absorber chip achieved high resolution and ultra-high sensitivity by sensing the changing values at maximum absorptions (A) and the shifting resonance frequencies (F) for the simulated detections of three typical Avian Influenza viruses (H1N1, H5N2, H9N2). In the following FDTD simulation with three selected virus models, each of the designed equivalent sample virus models revealed a sharp change of the maximum absorption in the absorption spectroscopy and resonance frequency, as shown in Figure 6. The maximum absorption, A (0.37, 0.44, and 0.42 THz), and resonance frequencies, F (0.36, 0.36, and 0.51 THz), of three common AI viruses are visibly altered when compared to the unloaded chip in Figure 6c,d. The author emphasized that the discerning contrast between ΔF and ΔA and the smaller virus size could enable the metamaterial chips to quickly, efficiently, and accurately detect, and real-time monitor AI viruses and future pathogens in the THz domain. The effect of thickness on the virus-contained solution with analyte virus H9N2 is shown in Figure 6e,f. Figure 6e depicts the biosensing frequency shift of absorption spectroscopy versus different analyte layer thicknesses, while Figure 6f depicts the frequency shift (ΔFth.) versus different analyte layer thicknesses, as ΔFth.

Planar THz plasmonic metasurfaces [59,60,61,62] of a toroidal dipole with maximum quality and capacity to detect specific nanoscale biomarkers would be an ideal technology for practical biomedical applications [63]. A toroidal dipole resonance is a plasmonic resonant mode based on magnetic currents and a part of the new family of non-radiative modes. It results when the magnetic moments of the current formed by incident radiation on a torus’s surface are aligned head-to-tail, form a dynamic vortex, and are well hidden by the radiation pattern of powerful classical EM multipoles because of the small far-field radiation signatures. Despite the difficulty in detecting toroidal excitations, well-designed metamolecules can improve toroidal dipole responsiveness. Electric and magnetic dipoles and quadrupoles must be subdued to achieve direct toroidal excitation, and the magnetic field inside the metamolecule must then be constrained into tight oscillatory loops. THz plasmonic biosensors based on toroidal resonance response have a huge impact and applicability in modern medical and therapeutic practices [64].

**Figure 7:**
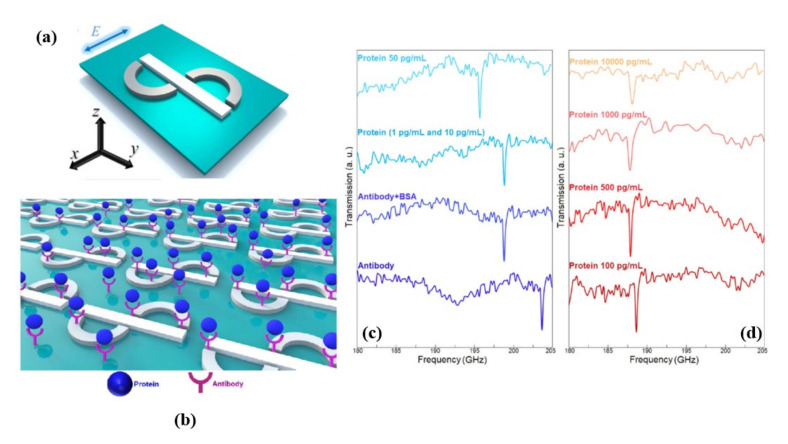
(**a**) Artistic perspective of compositional plasmonic resonators, (**b**) schematic demonstration of ZIKV envelope protein binding with respective antibody and transmission spectra for the toroidal resonant mode behavior for presence of different concentration of ZIKV envelope protein from (**c**) antibody to 50 pg/mL and (**d**) 100 pg/mL to 104 pg/mL. Reprinted with permission from [59]. Copyright 2017 American Chemical Society.

An artificially engineered multi-metallic planar asymmetric split type plasmonic resonators (as shown in Figure 7a,b) with iron (Fe, acts as magnetic resonator) and titanium (Ti, acts as electric resonator) components that allowed for toroidal dipole excitation in the terahertz (THz) domain with an experimental Q-factor of ~18 was reported in [59]. It was demonstrated that a room-temperature toroidal metasurface is a viable platform for immunosensing applications by using the plasmonic metasurface to detect Zika-virus (ZIKV) envelope protein (diameter~40 nm) using a specific ZIKV antibody as a proof of concept, taking advantage of the high-Q toroidal lineshape and its dependence on environmental perturbations. For relatively small concentrations (~pM), depending on the envelope protein of ZIKV the sharp toroidal resonant modes of the surface functionalized structures were shifted, as shown in Figure 7c,d. The findings of sensing studies showed that envelope proteins could be detected rapidly, accurately, and quantitatively, with a detection limit of ~24.2 pg/mL and a sensitivity of 6.47 GHz/log(pg/mL).

**Figure 8:**
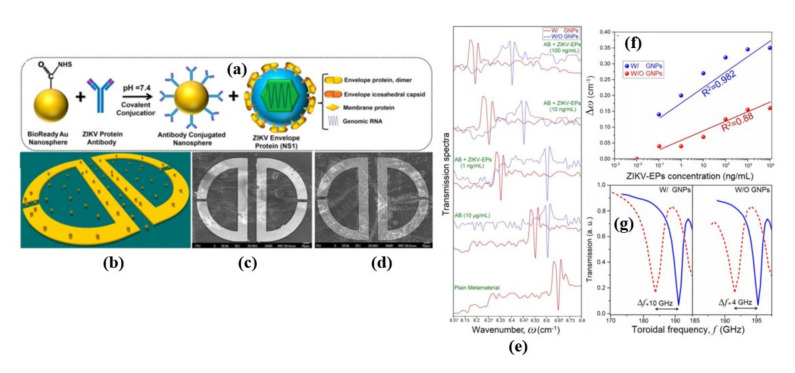
(**a**) Schematic flowchart of functionalized gold nanoparticle conjugation with ZIKV-AB and ZIKV-EPs NS1 with an explanation of the different parts. (**b**) Schematic representation of gold-nanoparticle-integrated toroidal unit cells. SEM images of a plasmonic metamolecule in the presence of (**c**) GNPs with AB and (**d**) ZIKV-Eps. (**e**) Transmission amplitude spectra for W/and W/O GNPs regimes in the presence of ZIKV-AB and ZIKV-EPs with different concentrations. (**f**) The toroidal resonance shift as a function of ZIKV-EPs concentration W/and W/O GNPs, and (**g**) the magnified transmission spectra as a function of frequency. Reprinted with permission from [63].

Sensitivity of the THz based metasurface was further improved by integrating the plasmonic metasurface and colloidal GNPs (gold nanoparticles) with an average of 40 nm diameter in a follow-up work [63], as shown in Figure 8a–d. Sensing and quantification of the ZIKV-EP in the assays was shown by monitoring the spectrum shifts of the toroidal resonances by changing the concentration (Figure 8e). Experiments with and without the gold particles were performed to demonstrate the impact of plasmonic GNPs in the metamolecules sensitivity enhancement (Figure 8e). 

The reproducibility, sensitivity, and limit of detection (LoD) of the planar toroidal THz metasensor (Figure 8f,g) were investigated by taking measurements of the shifts in toroidal dipolar momentum resonance frequency (up to Δω~0.35 cm^−1^) for varying concentrations of biomarker proteins. The results showed up to 100-fold sensitivity increments and could detect low molecular-weight biomolecules (~13 kDa) in diluted solutions, which establishes the platform as a highly promising, sensitive, and efficient THz metamaterial for biomarker, particularly virus, detection. For the GNP-integrated metasurface, the limit of detection (LoD) was ~560 pg/mL and sensitivity was 5.81 GHz/log(pg/mL), while for the bare metasurface, the limit of detection (LoD) was 12 ng/mL and sensitivity was 2.25 GHz/log(pg/mL).

**Figure 9:**
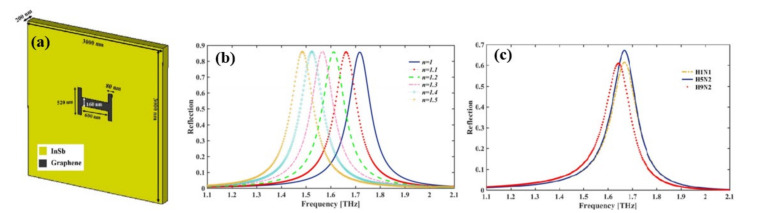
(**a**) Schematic illustration of the unit cell, (**b**) simulated reflection spectrum of biosensor in different mediums metamaterial reflector and (**c**) simulated results of reflectance spectra for different AI virus vs. frequency. Reprinted with permission from [65]. Copyright 2019 IEEE.

Depending on Fano-resonances a graphene-based H-shaped (Figure 9a) nanoscale metamaterial reflector [65] positioned at the middle of the InSb semiconductor film was reported for the detection of Avian Influenza (AI) viruses H1N1, H5N2, and H9N2. A resonant frequency shift following the virus deposition, a considerable value of RI sensing (RI FOM up to 2.86) and sensitivity up to 540 GHz/RIU (equivalent to 56.7 μm/RIU) confirms the applicability of the nano bio sensor in virus detection. The reflection spectra of the nanosensor are shown in Figure 9b with varying RI of the surrounding medium and the spectra blue shifted with decreased RI. The resonant frequencies for H1N1, H5N2, and H9N2 viruses are at 1.668, 1.665, and 1.641 THz, respectively, and the magnitude of the reflection is 61.4%, 67%, and 60.9% respectively (Figure 9c).

**Figure 10:**
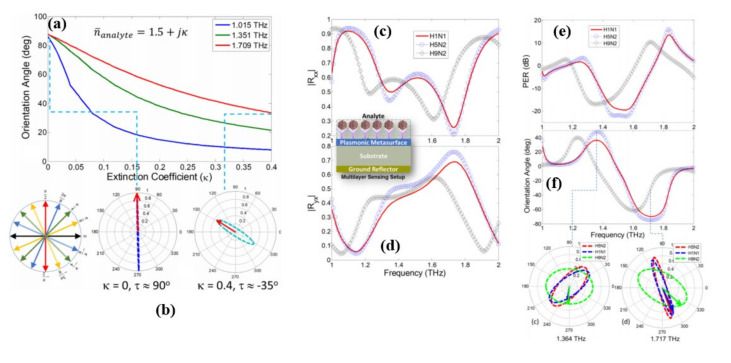
(**a**) Changes in orientation angle as κ is increased from 0 to 0.4 calculated at the frequencies where dominant power is reflected as cross-polarized fields. (**b**) contour path traversed by locus tip of electric field vector in time at fixed frequency of 1.7 THz for κ = 0 0 and κ = 0.4. (**b**) Magnitude response of (i) co-polarized (ii) cross-polarized reflection coefficients of the sensor for three different strains of Influenza viruses H1N1, H5N2 and H9N2. (**c**) PER and (**d**) Orientation angle spectrum for three different strains of influenza viruses H1N1, H5N2 and H9N2. The contour path traversed by locus tip of electric field vector in time at fixed frequency of (**e**) 1.364 THz and (**f**) 1.717 THz for all three strains of viruses. Reprinted with permission from [66]. Copyright 2021 Elsevier.

In [66], Amin et al. demonstrated a polarization-state sensing-based detection approach that can differentiate virus elements having optical properties that are very identical (Figure 10a,b). The polarization state sensing measurement setup comprises of a graphene-based plasmonic metasurface with chiral unit cells (Figure 10c,d). As a result, the polarization states of the reflected electric fields are particularly dependent on the refractive index of the dielectric surrounding the resonant unit cells. According to its complex dielectric constant of the viral analyte deposited on the metasurface its polarization state changes. Depending on the difference of the reflected polarization states, it is demonstrated that the proposed sensor can differentiate three influenza viruses that are quite similar: H1N1, H5N2, and H9N2 (Figure 10e,f). Because of its capacity to differentiate between analytes with identical optical properties based on the extinction coefficient (κ), we believe the suggested polarization-based sensing employing metasurfaces has great potential to separate viruses.

**Figure 11:**
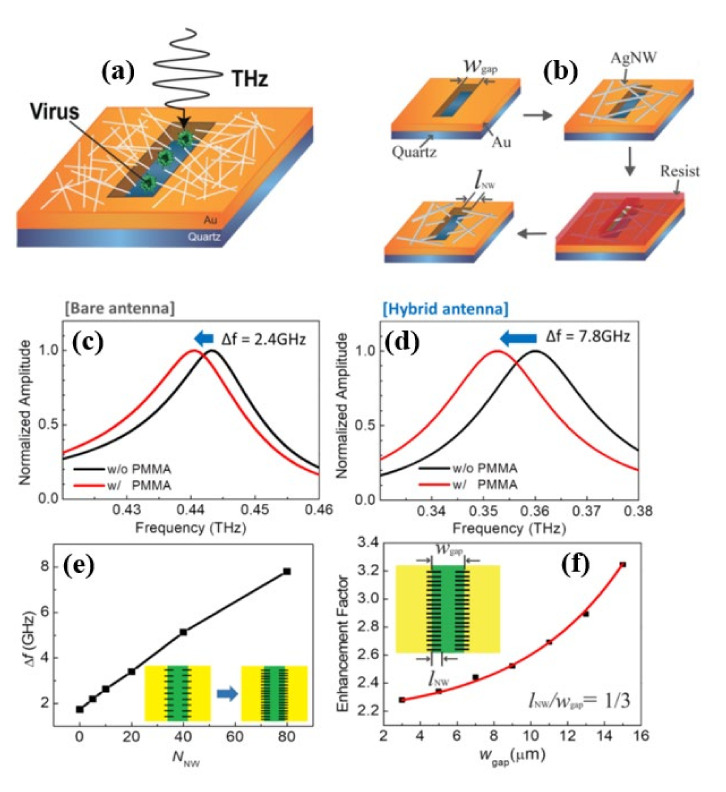
(**a**) Schematic of the THz hybrid slot antenna with protruding AgNWs. (**b**) fabrication processes for the THz hybrid slot antenna, (**c**) FDTD simulation transmission spectra through the bare slot antenna with (red) and without (black) PMMA [L = 200 μm and wgap = 15 μm]. (**d**) Transmission spectra through the hybrid slot antenna with (red) and without (black) PMMA for lNW = 5 μm. (**e**) Resonant-frequency shift as a function of the total number of AgNWs (NNW). (**f**) Enhancement factor as a function of wgap for fixed NNW = 80 and lNW/wgap = 1/3. Reprinted with permission from [67]. Copyright 2018 Springer.

Although nanogap metamaterials [42] was reported recently to improve virus detection limit below 100 nm using the nanolithography process, this fabrication process is difficult, costly, and time-consuming. As a result, one-dimensional nanowires and other novel functional materials with an inherent nanoscale dimension are preferred. In [67], silver nanowires (AgNWs) were used to improve the sensitivity of hybrid slot antenna designs for microbial detection in the THz frequency range without using the nanolithography approach as shown in Figure 11a,b. When poly (methyl methacrylate) (PMMA) and viruses were deposited, a resonant-frequency shift was observed. For a bare slot antenna (Figure 11c), resonant frequency exhibits a redshift of 2.4 GHz, whereas a hybrid antenna (Figure 11d) indicates a higher frequency shift of 7.8 GHz. Figure 11f shows Δf as a function of NNW, and it increases with the number density of AgNWs. Figure 11f shows that the sensitivity enhancement factor is a function of the width of the bare slot antenna. The sensitivity was studied by changing the slot antenna width and the density of the AgNW, and it was found to increase with AgNW density until saturation. An improvement of the sensitivity by a factor of more than 3.8 (~6.7 in terms of FOM) was reported for the 15 μm wide slot antenna. The devices were also used for PRD1 viruses resulting a 2.5 enhancement factor (3.4 in terms of FOM) for the 3 μm slot antenna width. Without using nanoscale fabrication methods, the study demonstrated a nanoscale detecting volume with substantial field enhancement.

**Figure 12:**
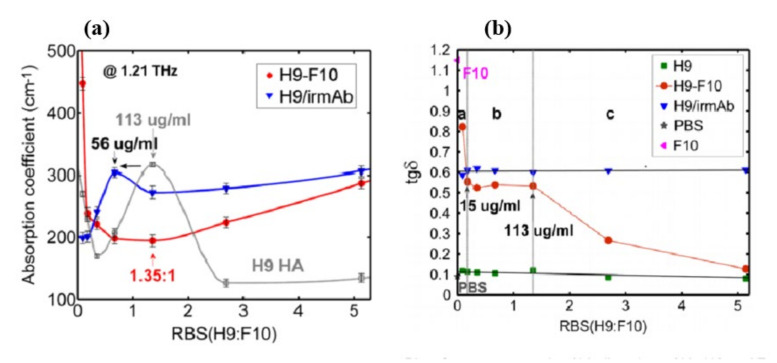
(**a**) Plot of tan δ versus ratio of binding sites of H9 HA and F10 and negative control, and H9 HA solutions. (**b**) Specificity and sensitivity measurements by ELISA. Reprinted with permission from [68]. Copyright 2015 SPIE.

Sun et al. [68], investigated the binding of the HA protein H9 to a HA-specific broadly neutralizing monoclonal antibody F10 using terahertz spectroscopy. In the liquid phase (~μm thickness), the absorption coefficient for three different samples: H9 HA with F10 (specific antibody), H9 HA, and H9 HA with irmAb (nonspecific antibody) was examined. For the three samples, the absorption coefficient vs. ratio of binding sites (which accounts for both protein and antibody concentrations) is shown in Figure 12a. It was discovered that whereas the hydration shell was always present in charged H9 HA and H9/irmAb solutions, the binding interaction between H9 HA and F10 nAb efficiently eliminated it (Figure 12a red solid line). The dielectric loss tangent provided a sensitive method of monitoring the H9-F10 interaction: the detection limit for H9 HA was 15 μg/mL, whereas the ELISA test was 113 μg/ml (Figure 12b). THz spectroscopy’s unique capabilities for exploring antibody–antigenbinding label-free are highlighted by the reported observations of H9 HA with a HA-specific broadly neutralizing monoclonal antibody F10.

**Figure 13:**
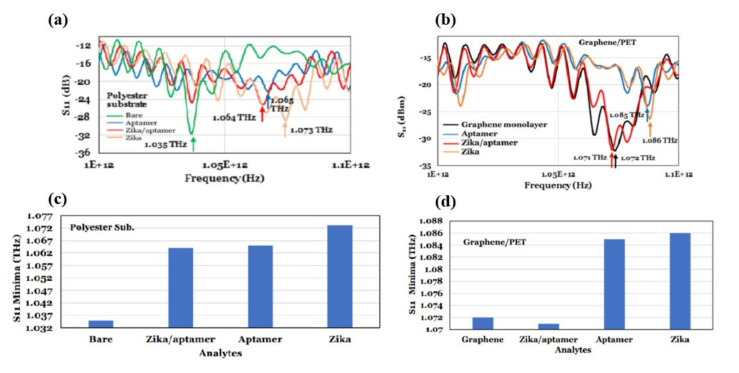
(**a**) Terahertz reflection (S11) properties of aptamer, aptamer/Zika, and Zika on polyester substrate. (**b**) Reflection spectra minima of different materials on polyester substrate. (**c**) Terahertz reflection coefficient of different aptamer and Zika on graphene. (**d**) Terahertz reflection minima for different analytes on graphene. Reprinted with permission from [69].

Terahertz reflection spectroscopy without metamaterial substrates was also used to detect Zika virus with and without a targeting/binding oligonucleotides (aptamers) [69]. For this study a heat inactivated Zika virus was used. The diameter and mass of the virus was 40 nm and 43 kDa (or 7.1 × 10–20 gm) respectively. In this study, the dielectric substrates were coated with the aptamer and the Zika virus contained pH-neutral buffer solutions in three different combinations for the reflection mode measurements: (i) a 70-µm spacer was utilized to keep the thickness of the Zika/aptamer consistent; (ii) its band gap energy was modified by employing a graphene layer to implement a reflection reference and increase the influence of analyte residual charge; and (iii) a polycarbonate substrate with 0.5 µm microbeads on Zika was used to investigate terahertz properties for various Zika concentrations. The Zika virus had a reflection minimum of 1.073 THz when using terahertz electromagnetic waves (0.75–1.1 THz), whereas Zika/aptamer complexes had a consistent terahertz reflection coefficient minimum of 1.064 THz. The minimum signal detected was of ~16 × 10^3^ Zika and the achieved sensitivity was 63 Hz/Zika by measuring the terahertz reflection from polyester microbeads coated with aptamers as a function of Zika concentration, as shown in Figure 13a,b. The Zika/aptamer combination seems to be performing as a dielectric while utilizing a monolayer of graphene on polyethylene terephthalate (PET), shifting the reflectance minimum from 1.072 GHz for graphene to lower frequency of 1.071 GHz for Zika/aptamer on graphene. As demonstrated in Figure 13c,d, Zika moved the minimum to 1.085 GHz, while aptamer moved it to 1.086 GHz. Different substrates, including 50 nm-gold films on polycarbonate, 30 nm thin glass slides, and Teflon were also investigated. The authors claim that their sensor could easily detect other viruses, such SARS-CoV-2, by employing specific aptamers or antibodies.

## 4. THz-Based Sensing and Detections for the Novel SARS-CoV-2

**Figure 14:**
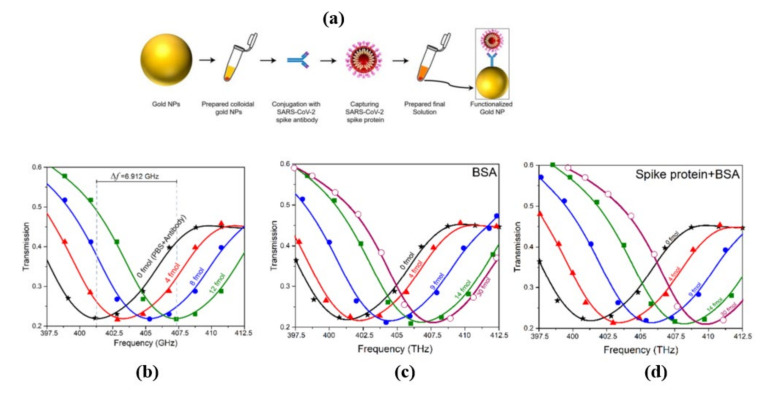
(**a**) Schematic workflow of the developed functionalized gold NPs conjugated with the respective SARS-CoV-2 antibody and spike proteins. (**b**) Measured transmission spectra of the THz metasensor device for different concentrations (4 fmol to 12 fmol) of SARS-CoV-2 spike protein. (**c**,**d**) Measured transmission spectra of the THz metasensor device for different concentrations (from 4 fmol to 30 fmol) of BSA and SARS-CoV-2 spike protein + BSA, respectively. Reprinted with permission from [70]. Copyright 2021 Elsevier.

Terahertz toroidal plasmonic metasurfaces can facilitate precise screening and detection of a variety of biomarkers through considerable field confinement at subwavelength geometries [59,63]. This property paves the way for the detection of targeted biomolecules at low concentrations in extremely diluted solutions. Ahmadivand et al. [70] used a toroidal plasmonic metasensor to sense COVID-19 spike protein at femtomolar levels. The reported sensor had a detection limit of ~4.2 fmol and a molecular weight of ~76 kDa, with a process time of around 80 min. The toroidal metasensor performed well at low concentrations, and the shift of the toroidal dipole resonance decreased dramatically and saturated with higher biomolecule concentrations (Figure 14). The feasibility of employing terahertz spectroscopy for early detection of COVID-19 was also investigated in [71]. It was demonstrated that viruses with diameters smaller than SARS-COV-2 virus (120 nm) such as PRD1 (60 nm), MS2 (30 nm), Avian Influenza Virus (80–120 nm), Zika Virus (50 nm) can be detected through terahertz technology-based biosensors possessing encouraging sensitivity. These approaches could also be effective to detect coronavirus, and more research into their utility in coronavirus detection is needed. Notable performance comparison of recent biosensors for virus detection are summarized in Table 1.

## 5. Challenges and Future Trends of THz-Based Sensing

The need for widely deployable technologies that can detect fatal infectious viruses has rapidly intensified, considering the pandemic events of the last two decades. Even though various molecular techniques (RT-PCR, RT-LAMP, etc.) and immuno-based techniques (ELISA, CLIA, etc.) are now available, they remain difficult and complex to deploy on a massive scale especially at point-of-care locations. Plasmonics biosensors also play a significant role in detecting fatal infectious viruses. So far, biosensing with high-quality factor plasmonic resonances has focused mainly on detecting spectrum shifts and their related intensity changes. The phase response of plasmonic resonances, on the other hand, has rarely been used because it necessitates a more complicated optical setup. Because of their great sensitivity and throughput potential, phase sensitive SPR sensors have attracted great attention in recent years [82,83]. The key goal of future development will be to increase the performance of phase sensitive SPR sensors in terms of sensitivity, dynamic range, spatial resolution, and portable and miniaturized sensor design. Phase-sensitive SPR sensors are expected to become more relevant in biological and chemical detection in the near future. 

Until now, THz biosensors primarily used spectrum or intensity modulation methods to estimate analyte concentration, despite the fact that phase and polarization states can provide additional information. An ultrasensitive THz biosensor has been theoretically shown in [84] using a passive bilayer metasurface to construct a two-resonator linked system that obeys passive parity time symmetry. By creating coupled resonators with orthogonal excitation orientations and retrieving the eigenfrequencies in polarization space, ultra-high THz sensitivity can be achieved by distinguishing eigenfrequency splitting even smaller than the resonance linewidth. According to electromagnetic simulations, the sensitivity of the analyte can reach up to 800 GHzRIU^−1^μm^−1^. In another study, a detector based on an orthogonally cross-nanowire network records the entire polarization state of terahertz pulses and demonstrates the viability of increased sensitivity response [85]. In this outline, THz technology has a strong potential for biosensing applications, especially for detecting deadly viruses or pathogens, and can complement or enhance existing approaches. Limitations of conventional THz-TDS in attaining sufficient sensitivity to monitor or differentiate the presence of viruses can be circumvented by employing engineered meta-material resonators. Meta- and nanomaterials with various unit cell geometries working in the THz region are an emerging option assuring much promise for on-site, high-speed virus detection and have the potential to go beyond the detection limit. Moreover, with recent advancements in miniaturized THz technologies [86,87,88,89,90], THz-based virus sensing platforms can be widely deployed, even to remote locations. Even though terahertz based biosensing methods have been used for several sectors [91,92], there is still a need for extensive research on SARS CoV-2 and similar virus detection. The SARS-CoV-2 pandemic is currently overtaking the globe. THz could be an excellent option to meet the pressing need for label-free, selective, repeatable, cost-effective, phase-sensitive, and rapid detection technologies to tackle this pandemic and the next one.

## 6. Conclusions

We have thoroughly reviewed different existing terahertz techniques and their sensing and detection efficiency for different viruses, including SARS-CoV-2 virus. Though terahertz technologies have not been fully explored for SARS-CoV-2 virus yet, there is a tremendous potential to develop sensor platforms by taking advantage of resonators with high Q-factors, ultra-tight field confinements, and small form factors. The presented THz approaches can lead to the development of rapid, inexpensive, and highly sensitive diagnostic tests for SARS-CoV-2 or other future pathological viruses. Further research is needed in this field to establish the benchmark for SARS-CoV-2 testing using terahertz technologies. Moreover, clinical validation of the developed technologies is important widespread adoption.

## Figures and Tables

**Table 1:** Performance comparison of recent biosensors for virus detection including SARS-CoV-2.

Concept of Work	Sensitivity/LOD	Detected Virus	References
THz toroidal metasensor	~4.2 fmol	SARS-CoV-2 spike protein	[70]
Dual-functional LSPR biosensor with Au Nanoislands	0.22 pM	SARS-CoV-2 spike protein	[72]
Gated graphene-enhanced FET based biosensor	1.6 × 101 pfu/mL (culture medium) 2.42 × 102 copies/mL (clinical samples)	SARS-CoV-2 spike protein	[73]
Au nanorod based plasmonics	111.11 deg/RIU	SARS-CoV-2 spike protein	[74]
Zr NPs with Zr QDs	79.15 EID/50 mL	Coronavirus	[75]
MIR Spectroscopic technique	95%	SARS-CoV-2	[76]
Raman Spectroscopic technique	83.7–97.5%	SARS-CoV-2	[77]
Thermoplasmonic-assisted dual-mode transducing (TP-DMT)	250 copies/mL	SARS-CoV-2	[78]
LSPR based opto-microfluidic sensing platform with gold nanospikes	0.08 ng/mL (~0.5 pM)	SARS-CoV-2	[79]
Carboxyl groups (PC)-coated magnetic nanoparticles (pcMNPs)	10 copy/mL	SARS-CoV-2 RNA	[80]
THz Slot antenna with silver nanowire	32.7 GHz·µm^2^/particle	PRD1	[67]
THz Toroidal metasensor	5.81 GHz/log(pg/mL)	Zika virus	[63]
THz Toroidal metasensor	6.47 GHz/log (pg/mL)	Zika virus	[59]
THz Au nano-antenna with 2D punctured rectangular slots	0.35 THz/RIU	H1N1, H9N2, H5N2	[49]
THz Rectangular Au metamaterial	80 GHz/particle/µm^2^	PRD1, MS2	[42]
THz SSPP Jerusalem Cross Aperture	0.5 THz/RIU	AIV	[53]
Hetero-assembled AuNPs sandwich-immunoassay LSPR chip	100 fg mL^−1^	Hepatitis B surface antigen	[81]

## Data Availability

Not applicable.
